# Revolutionizing neurotherapeutics: blood-brain barrier-on-a-chip technologies for precise drug delivery

**DOI:** 10.1097/MS9.0000000000001887

**Published:** 2024-03-04

**Authors:** Burhan Kantawala, Sanobar Shariff, Nagham Ramadan, Violette Fawaz, Youmna Hassan, Nadine Mugisha, Konstantin Yenkoyan, Abubakar Nazir, Olivier Uwishema

**Affiliations:** a Oli Health Magazine Organization, Research and Education; b Faculty of Global Surgery, University of Global Health Equity, Kigali, Rwanda; c Neuroscience Laboratory, Cobrain Centre; d Department of Biochemistry, Yerevan State Medical University named after Mkhitar Heratsi, Yerevan, Armenia; e Faculty of Medicine; f Faculty of Pharmacy, Beirut Arab University, Beirut, Lebanon; g Faculty of Medicine and Surgery, Ahfad University for Women, Omdurman, Sudan; h Department of Medicine, King Edward Medical University, Lahore, Pakistan

**Keywords:** blood-brain barrier, chip, delivery, drug, neurotherapeutics, neuro-vasculature

## Abstract

**Introduction:**

The blood-brain barrier (BBB) is a critical neurovascular unit regulating substances' passage from the bloodstream to the brain. Its selective permeability poses significant challenges in drug delivery for neurological disorders. Conventional methods often fail due to the BBB's complex structure.

**Aim:**

The study aims to shed light on their pivotal role in revolutionizing neurotherapeutics and explores the transformative potential of BBB-on-a-Chip technologies in drug delivery research to comprehensively review BBB-on-a-chip technologies, focusing on their design, and substantiate advantages over traditional models.

**Methods:**

A detailed analysis of existing literature and experimental data pertaining to BBB-on-a-Chip technologies was conducted. Various models, their physiological relevance, and innovative design considerations were examined through databases like Scopus, EbscoHost, PubMed Central, and Medline. Case studies demonstrating enhanced drug transport through BBB-on-a-Chip models were also reviewed, highlighting their potential impact on neurological disorders.

**Results:**

BBB-on-a-Chip models offer a revolutionary approach, accurately replicating BBB properties. These microphysiological systems enable high-throughput screening, real-time monitoring of drug transport, and precise localization of drugs. Case studies demonstrate their efficacy in enhancing drug penetration, offering potential therapies for diseases like Parkinson's and Alzheimer's.

**Conclusion:**

BBB-on-a-Chip models represent a transformative milestone in drug delivery research. Their ability to replicate BBB complexities, offer real-time monitoring, and enhance drug transport holds immense promise for neurological disorders. Continuous research and development are imperative to unlock BBB-on-a-Chip models' full potential, ushering in a new era of targeted, efficient, and safer drug therapies for challenging neurological conditions.

## Introduction

Highlights

- The blood-brain barrier (BBB) is a critical neurovascular unit regulating substances’ passage from the bloodstream to the brain. Its selective permeability poses significant challenges in drug delivery for neurological disorders. Conventional methods often fail due to the BBB’s complex structure.
- Microphysiological systems, employing microfluidic technology, intricately emulate the complex peripheral and central nervous systems, among other organs. These systems, encompassing organoids, three-dimensional (3D) printed tissues, and organ-on-a-chips, amalgamate in-vivo and in-vitro models, facilitating dynamic fluid flow within a 3D environment for precise tissue mimicry.
- For the treatment of neurological illnesses, the use of BBB-on-a-Chip models in drug delivery experiments shows great promise. Researchers may be able to create treatments for diseases that were previously thought to be challenging to target, such as Parkinson’s disease, Alzheimer’s disease, and brain tumours, by enhancing medication transport over the BBB.

**Figure 1:**
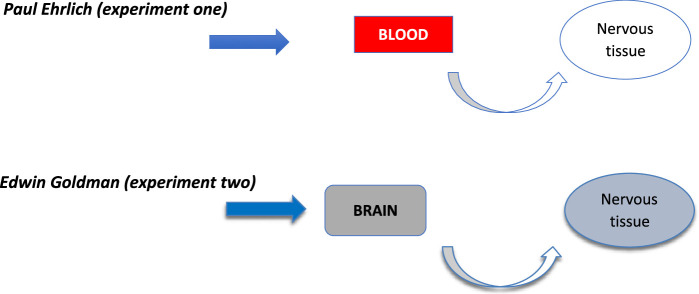
History of blood-brain barrier.

The central nervous system relies on the intricate biochemical processes involving macronutrients such as carbohydrates, fats, and proteins, as well as essential micronutrients including B vitamins and coenzymes for its sustenance and physiological stability. These processes are meticulously regulated by the blood-brain barrier, as illustrated in Figure 1 ^1^. This intricate barrier serves as a gatekeeper, controlling the influx of various compounds from the bloodstream into the brain tissue, thus safeguarding the delicate neural environment^2^.

The central nervous system is susceptible to pathological damage arising from infections, vascular diseases, immunological dysregulation, and degenerative processes, necessitating efficient therapeutic intervention^3^. Drug delivery, the method of administering medicinal compounds, is crucial for achieving therapeutic or preventive effects in humans. This process involves four fundamental stages: absorption, influenced by drug dosage and gut pH; distribution, affected by drug binding to plasma proteins, perfusion, diffusion; metabolism, regulated by enzyme systems; and excretion through organs such as the kidneys, lungs, saliva, and bile.

Drug delivery to the brain encounters a significant obstacle in the form of the blood-brain barrier (BBB). This barrier selectively permits entry only to lipophilic molecules with low molecular weight, rendering ~98% of pharmaceutical compounds unable to traverse it^4,5^. Enhancing this process holds paramount importance for several reasons. Physically, optimizing drug delivery enables smoother access to managing brain disorders by increasing drug distribution within the brain parenchyma. It allows for precise localization of drugs to specific sites of action, reducing systemic toxicity and enhancing treatment efficiency. Moreover, it mitigates the risk of clinical failure for potentially effective therapeutic agents. Psychologically, prolonged illness due to inefficient treatment correlates with an elevated risk of psychiatric disorders, hindering both the recovery process and overall quality of life. Neurodegenerative diseases, in particular, are linked to disruptions in emotions, cognition, and social behaviour^6^. Economically, inefficiencies in pharmacokinetic processes within brain tissues lead to protracted and financially burdensome treatment journeys. Therefore, improving drug delivery to the brain is inversely proportional to the duration and affordability of treatment.

This article aims to comprehensively review the existing literature pertaining to the development of a BBB on a chip for optimizing drug delivery to the brain.

## History

The origins of our understanding of the BBB can be traced back over a century ago. In a serendipitous turn of events, Nobel laureate Paul Ehrlich injected blue dye into mouse blood, leading to the revelation that the nervous tissue remained impermeable to the dye^7^. It was not until 50 years later that physician Edwin Goldman conducted a groundbreaking experiment, by injecting blue dye directly into the mouse brain, Goldman demonstrated that the nervous tissue indeed absorbed the dye, marking a pivotal moment in scientific inquiry^7^. This pivotal experiment laid the foundation for the concept of the blood-brain barrier, which is characterized as a selectively permeable barrier. It is composed of epithelial-like cells exhibiting high resistance tight junctions within the endothelium of capillaries.

## The BBB

The BBB stands as the largest interface governing the exchange between the bloodstream and the brain, acting as a vital neurovascular unit which is composed of distinct elements^8^:

### Endothelial cells

These cells form a monolayer of simple squamous epithelial cells lining the capillary walls. They are set apart from other vascular regions by the presence of tight junctions, luminal polarization, and a high abundance of mitochondria. Functionally, endothelial cells serve as a robust barrier, altering the physical properties of substances to control their passage.

### Astrocytes

These star-shaped cells envelop the surface of cerebral capillaries. They function as potassium channels, contribute to the maintenance of water homoeostasis, and regulate ionic concentrations. Astrocytes play a pivotal role in the intricate workings of the blood-brain barrier.

### Pericytes

Positioned between other cells, pericytes act as phagocytes, clearing foreign molecules and contributing significantly to maintaining the stability of the barriers. Their role is crucial in preserving the integrity of the BBB.

### Basement membrane

This membrane serves as the structural foundation, connecting the cells of the barrier. It plays a vital role in regulating communication between the intracellular and extracellular environments, ensuring the coordinated functioning of the components.

### Tight junctions

Representing the epitome of cellular barricades, tight junctions are apical cell-cell junctions characterized by their impermeability. These junctions consist of a group of proteins, including transmembrane proteins such as occludin and claudin-5, as well as various cytoplasmic proteins. Together, these components form a strong defense, allowing only select molecules to traverse the barrier, thus upholding the sanctity of the brain environment.

The BBB serves six critical functions: it maintains brain stability by safeguarding against pathogens and neurotoxins, ensures ionic homoeostasis and brain nourishment through specific ion channels and transporters, regulates neurotransmitters by guiding their influx, restricts plasma macromolecules from leaking into the brain—particularly controlling protein leakage to prevent neural apoptosis—and adjusts the permeability of cerebral capillaries. Additionally, it maintains brain volume by regulating the flow of water and salts^9^.The unique structure and functions of the blood-brain barrier pose three significant challenges for drug treatments targeting brain disorders^10^:

#### Structural challenge

The blood-brain barrier is characterized by a lipophilic plasma membrane allowing influx of only very small molecules and tight capillary junctions. These features, although obstacles, present opportunities for developing innovative drug delivery systems.

#### Chemical challenge

Multiple receptors, including transferrin and insulin receptors, are found on the surface of endothelial cells within the blood-brain barrier. Moreover, the barrier contains nuclear receptors expressing drug-metabolizing enzymes. Modulation of these receptors can enable drugs to traverse the barrier.

#### Transport-mediated challenge

The BBB features plasma membranes housing influx and efflux transporters that do not transport pharmaceuticals to the brain. Targeting these transporters offers a viable strategy to enhance drug delivery to the brain.

In response to these challenges, innovative methods and strategies have been devised for drug delivery to the brain. The current methodologies and their limitations are comprehensively documented and analyzed in Table 1 ^11^.

**Table 1:** Current methods and limitations in drug delivery

Method	Type	Advantages	Challenges	Limitations
Nanoparticles	Noninvasive	Brain targeting using specific physiological status	Structural	Neurotoxicity and cytotoxicity
Bypass BBB	Noninvasive	Bypass the BBB through intranasal, interstitial and intrathecal	3 CHALLENGES	Suitable for low dose only
Brain permeability enhancer	Noninvasive	Transiently open the BBB	Structural	Risk for human due to mismatch with rodents during clinical trials
Receptor mediated transcytosis, receptor antagonist and enzyme modulator	Noninvasive	High specificity, selectivity and affinity	Chemical	Toxicity
Carrier-mediated transport	Noninvasive	Ability to cross the BBB by intravenous injection	Transport mediated	Mainly for small molecules
Convection-enhanced delivery	Invasive	Reduces side effects to healthy brain tissue	3 CHALLENGES	Leakage of drug leading to toxicity

BBB, blood-brain barrier.

## Introduction to microphysiological systems (Organ-on-a-chip)

Microphysiological systems, employing microfluidic technology, intricately emulate the complex peripheral and central nervous systems, among other organs. These systems, encompassing organoids, three-dimensional (3D) printed tissues, and organ-on-a-chips, amalgamate in-vivo and in-vitro models, facilitating dynamic fluid flow within a 3D environment for precise tissue mimicry^12^. Notably, they replicate patient-specific pathology, disease initiation, and progression timelines^13^. These systems boast 3D structures mirroring human organs, ensuring physiologically relevant cell-cell and cell-matrix interactions, and facilitating real-time monitoring of disease progression and organ responses to drugs^14,15^. Pharmaceutical companies leverage these advantages to enhance drug discovery outcomes, improving accuracy and reducing costs by evaluating drug toxicity, safety, and efficacy^16^. Furthermore, given the brain’s intricate nature, microphysiological systems offer invaluable insights into neuronal transport and regeneration. Their 3D environment makes them ideal for simulating the physical and physiological complexities of the blood-brain barrier^17^. Despite challenges in differentiation protocols, recent strides in deriving brain endothelium from human-induced pluripotent stem cells (iPSCs) expand the array of brain cell types available for clinically relevant models in central nervous system diseases^18,19^ (Table 2).

**Table 2:** A comparative analysis of physiological and anatomical design consideration, cells and biomaterial, and their usage in the nervous system

System	Physiological and anatomical design consideration	Cells and biomaterial	Usage	Refs
Microvasculature	Endothelium lining BBB using fluidic shear stress Blood vessels using fluid shear stress	Cells: HUVECs, hCMEC/D3 Fabric: Microfluidic Cells: HUVECs/mouse fibroblasts/mouse smooth muscle cells Fabric: microextrusion bioprinting, Microfluidic	Drug testing Drug testing System development	^20,21^
Brain	Neural tissue Blood-brain barrier	Cells: 1-rat embryonic neurons and astrocytes,mouse neural stem cells, mouse primary cortical neurons 2-hESCs, hPSCs Fabric: 1-microextrusion bioprinting 2- microwell array Cells: human limbal cells Fabric: human amniotic membrane Cells: endothelial/ astrocytic cell lines; hBMVEC/pericytes/astrocytes Fabric: Microfluidics	System development and drug testing Drug testing	^22,23^ ^24^
Eye	Corneal epithelium Corneal stroma using cell alignment Corneal endothelial layer using mechanical properties full-thickness cornea	Cells: human limbal cells Fabric: human amniotic membrane Cells: human and rabbit corneal fibroblasts, hCSSCs/hCFs Human keratocytes Fabrics: silk microgrooves; microextrusion bioprinting Cells: primary human corneal endothelial cells, human, sheep or bovine corneal endothelial cell lines Fabric: hydrogel substrates rabbit corneal epithelial cells/keratocytes/endothelial cells hESC-derived limbal epithelial cell-like cells/corneal endothelial cell-like cells Fabric: 1- fibrin-agarose hydrogels 2- decellularized porcine cornea	Epithelium transplantation System development System development Cornea transplantation System development Cornea transplantation	^25^ ^26,27^ ^28,29^ ^30,31^
Tumour	Vascular interface using fluidic shear stress Stromal interaction using fluidic shear stress Glioblastoma using oxygen gradient	Cells: HDMEC/ breast cancer cells HUVECs, lung fibroblasts, monocytes, melanoma cancer cells Bone marrow stromal cells/ liver tumour cells Patient-derived glioblastoma and vascular endothelial cells Fabric: microfluidics, microextrusion bioprinting	Drug testing and system development	^32–35^

BBB, blood-brain barrier; Refs, references.

## Design and construction of BBB-on-a-chip

The organ-on-a-chip technique integrates in-vivo and in-vitro models, enhancing physiological microenvironments, imaging systems, and real-time sensor outputs^36^. To replicate the human BBB, models are exposed to physiological fluid flow, establishing realistic dimensions. Various BBB models include static, Dynamic in-vitro model (DIV), and BBB-on-a-chip models. BBB-on-a-chip models, which employ microfluidic technology, have excelled, considering blood flow effects and enabling specific molecule transport screening^37^. These models reconstruct tight junctions in monoculture and co-culture settings, incorporating endothelial cells with astrocytes and pericytes in 2D and 3D microenvironments^24,38^.

Microvasculature endothelial cells from animals or humans are subjected to physiological shear stress. BBB models on chips also allow the study of complex mechanisms for leucocyte recruitment^39^. Additionally, the glymphatic pathway, clearing brain solutes potentially linked to neurodegenerative diseases, can be intensively studied using microfluidic devices, applying physiologically relevant blood pressure, intracranial pressure, and flows^40,41^. Transepithelial electrical resistance (TEER) serves as a quick, noninvasive measure of brain tightness, comparable between devices. Recording electrical and biochemical signals of sensory neurons presents challenges, addressed using multiple microelectrodes and compatible microimaging systems on PDMS chips. Microfluidic combined microelectrodes facilitate subcellular structure visualization and neuronal electrical activity measurement on BBB chips^42^ (Table 3).

**Table 3:** Different types of BBB-on-chip models

Types of BBB models	Materials and components	Cell types	Culture techniques	Application and limitation	Refs
Vertical 2D culture	PDMs	HBMEs, pericytes, astrocytes, hiNPCs	PC	Modelling of the BBB function and drug testing ( toxicity) and permeability of CNS	^43^
Parallel 3D chambers	PDMS	RBE4 cells and astrocytes	Pores generated by lithography between two chambers	Intergration of human BBB microfluidic model into a high-throughput plate-based format for drug screening purposes	^44^
Static 2D models (*in vitro* )	PLGA nanofiber mesh Collagen gel covered with monolayer of brain microvascular endothelium	HIPSC-EC and astrocytes Brain endothelial cells Pericytes and MSCs IPSC-BMECs, astrocytes, pericytes and neurons	Polymer trans well membrane model Membrane free hydrogel BBB model	Human BBB physiology and pathology with higher TEER value and good barrier functions Nanoparticule transcytosis quantification and transendothelial PEG-P (CL-g-TMC)polumersomes delivery. BBB phenotypes with TJ and permeability and up-regulating pericytes mark.	^45^ ^46^ ^47^ ^48^
3D biomimetic multichannels culture	NA	BEnd.3, U87 gliobalstoma cells	PC	Formation of a 1:1 scale biomimetic BBB model with satisfied TEER and capability for drug screening.	^49^
Parallel 3D multichannels culture	PDMS	hUVEC, rat astrocytes in gel, rat neurons in gel	NA	New platform for the development of a more sophisticated and complex 3D in-vitro neurovascular model and has good observation of neurons.	^50^
2D vertical tandem multi chamber	PDMS	HBMECs, astrocytes, pericytes	PC	Mimicking the effects of intravascular administration of the psychoactive drug methamphetamine and determines metabolic coupling between the BBB and neurons.	^51^
DIV-model	Transmural microholes	Astrocytes and hECs PBMECs	3D vasculogenic model QV-600 chamber multi chamber perfusion system	Studies BBB Enhance and maintain TEER for longer Used to investigate penetration of anti-epileptic drugs.	^52^ ^53^ ^54^
Microfluidic 3D model	Collagen I gel	ECs, astrocytes and pericytes	3D vasculogenic hydrogel model	Simple, new, cost effective in-vitro model for targeting neuroinflammatory conditions	^55^

2D, two dimensional; 3D, three dimensional; BBB, blood-brain barrier; BMEC, brain microvascular endothelial cell; CNS, central nervous system; EC, endothelial cell; h, human; hiPSC, human-induced pluripotent stem cell; iNPCs, induced neuron progenitor cells; m, mouse; NA, not applicable; NSC, neuron stem cell; PC, polycarbonate; PDMS, polydimethylsiloxane; PET, polyethylene terephthalate; r, rat; TEER, transepithelial electrical resistance; UVEC, umbilical vein endothelial cords.

### Mimicking BBB properties recapitulating BBB-specific features

Mimicking the intricate qualities of the BBB involves replicating its unique characteristics, such as tight junctions between endothelial cells acting as a physical barrier, specialized transporters controlling molecule transit, enzymatic metabolism of certain chemicals, and complex cellular signalling processes regulating permeability^56,57^. Researchers utilize cutting-edge models like BBB-on-a-Chip systems and co-culture approaches to replicate these BBB features, enabling the creation of specialized treatments for neurological illnesses and enhancing understanding of drug interactions with the BBB.

### Testing and validation of BBB-on-a-chip models

The development of BBB-on-a-Chip models offers a potential strategy for imitating BBB features^37,58^. These microfluidic systems incorporate cells, tissues, and biomimetic components, mimicking the fundamental properties of the BBB^59^. Essential steps in evaluating these models involve rigorous testing and validation. Researchers assess the permeability of the Brain-on-a-Chip to various compounds, including medicines and nanoparticles, to gauge its resemblance to the in-vivo BBB^60^. Furthermore, they evaluate the model’s receptivity to diverse stimuli and its ability to reproduce unique traits specific to particular diseases. This evaluation process is crucial for researching neurological disorders and conducting medication screening^61,62^.

### Advantages of BBB-on-a-chip models over traditional in-vitro and in-vivo models

Compared to traditional in-vitro and in-vivo models, BBB-on-a-Chip models offer numerous advantages^63^. They enhance drug behaviour prediction at the barrier, increase physiological relevance by simulating dynamic cell interactions within the BBB, and mitigate ethical concerns associated with animal testing. Additionally, these models are more economical and resource-efficient, addressing ethical issues and significantly reducing research timeframes^64^. In conclusion, BBB-on-a-Chip models present a promising method for simulating BBB characteristics. They offer substantial precision, effectiveness, and ethical considerations in neuroscientific and pharmaceutical research, ultimately advancing our comprehension of the blood-brain barrier and neurological diseases^37,65^ (Table 4).

**Table 4:** Comparison between traditional in-vivo models, traditional in-vitro models, and BBB-on-a-chip models in different aspects

Aspect/areas	Traditional in-vitro models	Traditional in-vivo models	BBB-on-a-chip models
Physiological relevance	Limited capacity to accurately imitate BBB physiology.	Although in-vivo models frequently mimic the BBB, they might not accurately reflect human biology.	High quality replication of BBB features, such as tight junctions, transporters, and cellular connections.
High throughput	Slower and less suited to high-throughput screening.	Expensive and time-consuming, particularly for animal investigations.	Allow for the testing of numerous drug candidates in parallel, speeding up the drug discovery process.
Real-time monitoring	Most conventional in-vitro models have a limited ability for real-time monitoring.	In-vivo real-time data collecting is feasible but could be intrusive and complicated.	Real-time evaluation of drug transport and BBB responses with the provision of dynamic data.
Disease modelling	Accuracy in reproducing illness conditions is limited.	Diseases can be replicated in in-vivo models, although they may be difficult to modify or control.	Can be used to examine drug distribution in the setting of neurological illnesses and add disease-specific components.
Ethical considerations	Involves using animals, which raises ethical issues and regulatory difficulties.	Involves animal testing, which might lead to logistical and ethical issues.	Reducing the necessity for animal testing will help to address ethical issues.
Cost-effectiveness	The price may vary, but it might be less than for in-vivo experiments.	Frequently more resource-intensive, including upkeep and care for animals.	Cost-effective because fewer resources are needed.

BBB, blood-brain barrier.

## Drug delivery optimization

### How BBB-on-a-chip enhances drug delivery studies

Drug delivery studies have been completely transformed by BBB-on-a-Chip models, especially in the context of neurological illnesses. For enhancing drug delivery, these microfluidic devices, which mimic the BBB, provide numerous benefits: (Table 5).

**Table 5:** Advantages/usages of microfluidic devices (BBB-on-a-chip models) in different areas

Areas	Advantages/usage
Physiological relevance	Tight connections between endothelial cells and the existence of transporters are two distinguishing characteristics of the BBB that BBB-on-a-Chip models accurately mimic^62,66^. Because of this physiological relevance, scientists/researchers may examine how medications interact with the barrier in a controlled setting, giving them information about whether they can pass the BBB^67^.
High throughput	Potential drug candidates can be screened in a high-throughput manner using these models. Multiple substances can be tested at once by researchers hastening the drug development process^68^.
Real-time monitoring	Drug transport and cellular responses can be observed in real-time using BBB-on-a-Chip technologies, hence, this dynamic examination offers insightful information on medication permeability and potential BBB side effect^69–71^.
Disease modelling	These models allow for the incorporation of disease-specific components, enabling researchers to examine drug delivery in the setting of neurological diseases like Alzheimer’s or glioblastoma. This aids in identifying prospective treatments and evaluating their effectiveness^71–73^.

BBB, blood-brain barrier.

### Case studies demonstrating improved drug transport

The increased drug transport abilities of BBB-on-a-Chip models are highlighted in several case studies. For instance, researchers have tested potential treatments for brain tumours using these systems and have seen better drug penetration through the BBB, resulting in more successful therapies^69,74^. Additionally, BBB-on-a-Chip models have contributed in the discovery of compounds that can improve medicine delivery to the brain, potentially reducing the progression of neurodegenerative illness^75,76^.

### Potential impact on neurological disorders

For the treatment of neurological illnesses, the use of BBB-on-a-Chip models in drug delivery experiments shows great promise. Researchers may be able to create treatments for diseases that were previously thought to be challenging to target, such Parkinson’s disease, Alzheimer’s disease, and brain tumours, by enhancing medication transport over the BBB^77,78^. The use of medications that are specifically targeted to afflicted brain regions while sparing healthy tissue may result in more effective therapy with fewer adverse effects.

In the context of neurological illnesses, BBB-on-a-Chip models have opened up new opportunities for drug delivery optimization. They may speed up the development of novel therapies by simulating BBB features and enabling high-throughput screening, thereby enhancing the prognosis for patients with difficult neurological disorders.

## Addressing challenges and future directions

Despite significant progress, challenges persist in the utilization of BBB-on-a-chip, hindering its widespread adoption. Accurately replicating the intricate BBB within microfluidic chips remains a challenge. Commonly used Polydimethylsiloxane (PDMS) cannot combine with hydrophobic compounds, impacting drug concentration and efficacy. Addressing this, thermoplastic-based chip fabrication emerges as an optimal solution due to superior compatibility with biomimetic materials, enhanced stability, light permeability, and electrical conductivity^78^. Economical concerns also loom large. Scaling up production and ensuring cost-effectiveness for widespread use in pharmaceutical research and clinical applications demand attention. While PDMS is prevalent in academic research, its unsuitability for large-scale commercial production necessitates exploration of alternatives like thermoplastics, despite their manipulation challenges^65^.

Preserving BBB-on-a-chip stability and consistency is paramount. Replicating mechanical forces influencing BBB functions, such as pulsatile blood flow, cues from neighbouring tissues, and shear stress, remains complex yet vital for accurate drug delivery optimization. Human-induced pluripotent stem cells (iPSCs) offer a promising solution. They generate Brain Microvascular Endothelial Cell (BMEC)-like monolayers expressing tight junctions and BBB-related markers. Further differentiation protocol modifications could yield transcriptomically closer iBMECs, enhancing BBB modelling. iPSC-based BBB-on-a-chip exhibits physiologically significant Transepithelial/Transendothelial Electrical Resistance (TEER) values, crucial for maintaining BBB function under elevated shear stress^79^.

Emerging technologies, such as advanced nanoparticle formulations and biomimetic nanoparticles, are revolutionizing drug transport across the BBB^80,81^. Noninvasive modulation through focused ultrasound techniques^82^ and cutting-edge imaging methods for real-time monitoring^83^ and assessing novel delivery methods^84^ further enhance BBB-on-a-chip capabilities. Interdisciplinary collaborations among pharmacology, biology, engineering, and materials science experts are pivotal in designing and optimizing these microfluidic systems^85^. BBB-on-a-chip holds tremendous potential to enhance drug delivery to the brain, addressing various neurological conditions and central nervous system diseases^86^.

### Limitations

The study encountered several noteworthy limitations. The authors faced logistical challenges arising from disparate geographical locations, diverse time zones, varying educational backgrounds and degrees, as well as distinct work responsibilities. These inherent differences impeded the scheduling and participation in discussion meetings, thereby affecting the efficiency of follow-up and revision processes. Additionally, the insufficient number of articles pertinent to the research topic posed a challenge, as it fell short of the targeted coverage.

The study content faced limitations aligned with the three specific objectives of this review paper. Firstly, in addressing the BBB and the associated drug delivery process, challenges were encountered in the creation of a visually impactful illustration elucidating the intricate structure of the BBB. Moreover, the extensive array of methods for drug delivery to the brain led to a somewhat congested presentation of data. Secondly, while exploring organ-on-chip technologies, limitations arose from the difficulty in providing a concise explanation of the prior applications of this technology due to the scarcity of comprehensive research papers on the topic. Thirdly, in outlining the drug delivery optimization process through the BBB on a chip, limitations emerged during the validation of its significance through case studies, with only four studies identified, resulting in a less robust justification for the intervention. Additionally, the broad scope of discussion regarding the validation of BBB-on-a-chip models presented challenges.

We recommend several directions for further exploration of this topic. These include a heightened focus on experimental research and case studies, a dedicated investigation into the efficacy of BBB-on-a-chip in neurodegenerative diseases, and an enhancement of the scope of discussion through the incorporation of illustrative elements and straightforward justifications.

## Conclusion

The advent of BBB-on-a-Chip models marks a pivotal breakthrough in drug delivery research. These microphysiological systems faithfully replicate the intricacies of the BBB, providing a controlled setting to study drug interactions and assess medication permeability in neurological disorders. Unlike traditional in-vitro and in-vivo models, BBB-on-a-Chip models confer notable advantages. They facilitate high-throughput screening, swiftly evaluating multiple drug candidates, and offer real-time monitoring of drug transport and cellular responses, yielding invaluable insights into medication delivery. This innovative approach not only deepens our understanding of neurological disorders but also heralds targeted, more efficient, and safer drug therapies for challenging conditions like Parkinson’s disease, Alzheimer’s disease, and brain tumours.

Rigorous testing and validation are imperative to ensure the accuracy and reliability of BBB-on-a-Chip models. Researchers meticulously evaluate these models’ permeability to various compounds, replicating real-world conditions. Through meticulous testing, scientists can assess the models’ responsiveness to stimuli and disease-specific traits, augmenting their utility in neurological research and drug screening. While BBB-on-a-Chip models exhibit astounding potential, persistent challenges need addressing. Accurately replicating the BBB’s complexity within microfluidic chips, ensuring stability and consistency, and tackling economic concerns are pivotal tasks. Additionally, emerging technologies like advanced nanoparticles, focused ultrasound techniques, and cutting-edge imaging methods present new avenues for exploration. Interdisciplinary collaborations among experts in diverse fields are imperative for optimizing these microfluidic systems, paving the way for groundbreaking advances in brain drug delivery.

**Figure 2:**
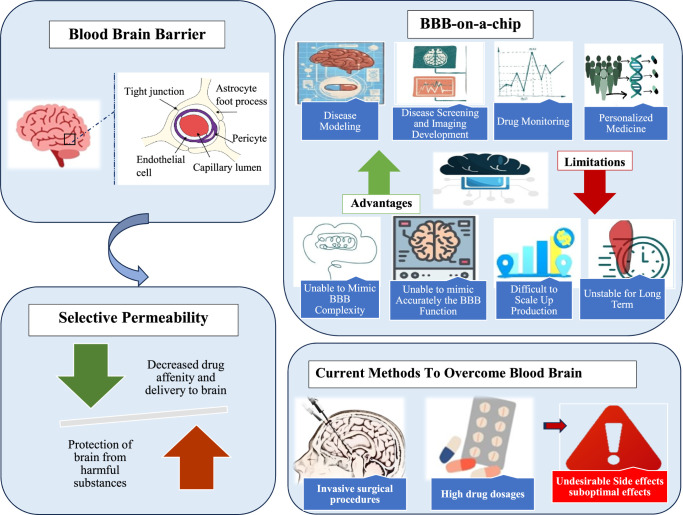
Summary of the various key points discussed in the manuscript.

As these promising advancements unfold, a compelling need for ongoing research and development becomes apparent. Collaborative efforts across scientific disciplines are vital to refining BBB-on-a-Chip models, overcoming challenges related to replicating BBB complexity, and ensuring long-term stability. This call for continuous research underscores the urgency in unlocking the full potential of BBB-on-a-Chip models, seamlessly integrating them into mainstream drug delivery strategies, and significantly improving patient outcomes in the realm of neurological diseases (Fig. 2).

## Ethics approval

Not applicable.

## Consent

Informed consent was not required for this article.

## Source of funding

We have not received any financial support for this manuscript.

## Author contribution

All authors have approved the final manuscript for submission. B.K.: supervising the draft, reviewing and editing, project administration. O.U.: conceptualization, writing-review and designing, project administration. S.S.: writing the first draft and revising. N.R.: writing the first draft and revising. V.F.: writing the first draft and revising. Y.H.: writing the first draft and revising. N.M.: writing the first draft and revising. K.Y.: writing the first draft and revising.

## Conflicts of interest disclosure

The author declared no conflicts of interest.

## Research registration unique identifying number (UIN)

Not applicable.

## Guarantor

Abubakar Nazir.

## Data availability statement

Not applicable.

## Provenance and peer review

Not commissioned, externally peer-reviewed.
